# Plastic Changes in the Spinal Cord in Motor Neuron Disease

**DOI:** 10.1155/2014/670756

**Published:** 2014-04-16

**Authors:** Francesco Fornai, Michela Ferrucci, Paola Lenzi, Alessandra Falleni, Francesca Biagioni, Marina Flaibani, Gabriele Siciliano, Francesco Giannessi, Antonio Paparelli

**Affiliations:** ^1^Department of Translational Research and New Technologies in Medicine and Surgery, University of Pisa, Via Roma 55, 56126 Pisa, Italy; ^2^IRCCS Neuromed, Via Atinense, 86077 Pozzilli, Italy; ^3^Department of Clinical and Experimental Medicine, University of Pisa, Via Roma 55, 56126 Pisa, Italy

## Abstract

In the present paper, we analyze the cell number within lamina X at the end stage of disease in a G93A mouse model of ALS; the effects induced by lithium; the stem-cell like phenotype of lamina X cells during ALS; the differentiation of these cells towards either a glial or neuronal phenotype. In summary we found that G93A mouse model of ALS produces an increase in lamina X cells which is further augmented by lithium administration. In the absence of lithium these nestin positive stem-like cells preferentially differentiate into glia (GFAP positive), while in the presence of lithium these cells differentiate towards a neuron-like phenotype (**β**III-tubulin, NeuN, and calbindin-D28K positive). These effects of lithium are observed concomitantly with attenuation in disease progression and are reminiscent of neurogenetic effects induced by lithium in the subependymal ventricular zone of the hippocampus.

## 1. Introduction

Plastic changes were described in the spinal cord in the course of amyotrophic lateral sclerosis [1, 2]. These consist of various morphological effects involving both resident cells such as neurons and glia and nonresident inflammatory cells. Among plastic effects, the sprouting of axon collaterals from spared motor neuron at the level of the peripheral muscles is well described [3, 4]. This is consistent during the disease course and it represents a marker of disease when it is detected by using electromyography. On the other hand, neuronal plasticity involving the motor neuron cell body in the ventral horn of the spinal cord represents a critical issue, and it is a hot topic in ALS research. In detail, the lack of effective neurogenesis in the course of ALS provided the basis to plan stem cell transplantation in humans either to substitute or prolong the survival of spared motor neurons [5, 6]. In keeping with stem cells, only a few studies focused on the occurrence of adult stem cells in ALS spinal cord. These studies documented the occurrence of increased cell proliferation during experimental models of spinal cord disease, mostly following traumatic spinal cord injury ([7] for a comprehensive review). Only a few studies analyzed endogenous spinal cord stem cells during ALS [8–11] and being the most recent studies rather focused on detailing the transplantation of exogenous stem cells previously induced to differentiate towards a neuron-like phenotype [12–16]. In fact, despite the fact that spinal cord disorders increase proliferation of neural progenitor cells (NPC), these generate preferentially glial cells instead of neurons [9, 10, 17] according to the concept that spinal cord environment has a strong gliogenic influence [7]. Thus, in order to address NPC proliferation towards a neuronal phenotype, further stimuli in the course of the disease need to be provided. It is well known that adult stem cells in both humans and various animal species* in vivo* are induced to proliferate and differentiate following lithium administration. This effect is well established in the subependymal ventricular zone (SVZ) of the lateral ventricles where lithium dramatically increases the amount of neurogenesis towards a specific calbindin-D28K neuronal phenotype [18]. In previous studies we found that lithium increases the amount of calbindin-D28K positive cells in the ventral horn of the spinal cord of G93A mice [19, 20]. Most of these calbindin-D28K positive cells costain for BrdU [19–21]. Adult stem cells in the spinal cord appear in the ependymal (and subependymal) zone within lamina X. In fact, as scholarly reviewed by Sabelström et al. [7], studies based on mapping the genetic fate and isolation by flow cytometry indicate that ependymal cells in the adult spinal cord own stem cell potential [22–24].

Therefore, in the present study, we analyzed lamina X around the central canal including the ependymal layer which possesses stem cell-like activity in G93A mice with or without chronic lithium administration.

In detail, we measured the number of lamina X cells as well as the presence of specific phenotypes such as nestin, GFAP, *β*III-tubulin, NeuN, and calbindin-D28K. These cell counts were carried out at the terminal disease stage when the occurrence of protective effects by lithium was documented as both increased survival and motor performance.

## 2. Materials and Methods

### 2.1. Animals

Male B6SJL-TgN(SOD1-G93A)1Gur mice, expressing the human G93A Cu/Zn superoxide dismutase 1 (SOD1) mutation (*n* = 10) and related wild type (WT) littermates (*n* = 10) were purchased from the Jackson Laboratory (Bar Harbor, ME, USA) via Charles River (Calco, LC, Italy). Mice received food and water* ad libitum* and were housed under controlled conditions: 12 hours light/dark cycle and at 21°C room temperature.

### 2.2. Experimental Groups and Treatments

Transgenic G93A superoxide dysmutase 1 (SOD) mice and their wild type (WT) littermates were divided into four experimental groups: lithium-treated G93A mice (*n* = 5); saline-treated G93A mice (*n* = 5); lithium-treated WT mice (*n* = 5); saline-treated WT mice (*n* = 5). Lithium chloride (Sigma, St. Louis, MO, USA) was administered (to both G93A and WT mice) every other day at the dose of 1 mEq/Kg i.p. dissolved in saline (sodium chloride 0.9%) in a volume of 200 *μ*L. Controls (both G93A and WT mice) received an equal volume of saline. All treatments were carried out in the morning, between 9.00 and 12.00 am. Lithium was administered starting at 67 days of age until the end point of disease. In order to avoid arbitrary assumptions we specifically considered “end point” the time point when the mouse was no longer able to right itself from a sided position during a 30-second time interval (due to a severe palsy of all limbs), according to Parone et al. [26]. Survival was considered until this end point and it was plotted as a Kaplan-Meier curve. When the genetic disease eventually led to the end point each mouse was sacrificed in order to avoid discomfort due to impaired feeding, drinking, and, as originally reported [27], also breathing. Sacrifice occurred by using deep chloral hydrate anaesthesia while perfusing the mouse to preserve the spinal cord for light microscopy. All experiments were carried out in compliance with the European Council directive (86/609/EEC) for the use and care of laboratory animals.

### 2.3. Behavior

Each behavioral test was carried out by gently handling each mouse. All motor tests began at 60 days of age, one week before starting chronic lithium administrations, and they were performed weekly for all animal groups (*n* = 5 per group). Locomotor activity, motor strength, and motor coordination were evaluated as described in previous works [19, 28] by using stride length test, paw grip endurance (PaGE) test, and rotarod test, respectively. Each test was scored blindly.

### 2.4. Stride Length Test

The stride length was measured following the method by Fernagut et al. [29] which was further modified by Fulceri et al. [30]. Briefly, the apparatus was included into an open field (80 × 80 × 30 cm). In this open field a runway wide illuminated (75 × 5 cm) was leading into a dark box (20 × 15 × 10 cm). Each mouse was allowed to run on the bright runway towards the dark box. The hind paws of each mouse were ink-painted and the distance between two paw prints was counted as the stride length. The three longest stride lengths were selected from each test. Data are reported as the mean of these stride lengths. Mice unable to walk were scored zero.

### 2.5. Paw Grip Endurance (PaGE) Test

The PaGE test was used to assess the motor strength as reported originally by Weydt et al. [31]. Each mouse was placed over a meshed wire grid, which was shaken to force the mouse to grip the grid. Then the grid was gently turned upside down and the latency until a mouse was able to keep the hold was recorded with a cutoff time of 90 seconds. Each mouse was scored for three consecutive trials and the longest latency was recorded. Animals unable to grip the grid were scored zero.

### 2.6. Rotarod Test

Motor coordination was evaluated by a rotating rod. The rod was automatically rotating at 15 rpm and the time during which the mouse was able to stay on the rod during a 10-minute (600 seconds) interval was recorded. The best result of three trials was recorded for each mouse.

All behavioral data are expressed as the mean ± SEM from each group for each unit of measurement, which was used in each test. When showing the data we plotted only the symptomatic groups of mice (G93A) treated with either saline or lithium. Inferential statistics were applied comparing these two groups of mice using *t*-test for continuous values with normal distribution. The null hypothesis *H*_0_ was rejected for *P* ≤ 0.05.

As an additional validation of inferential statistics, motor behavior, apart from using ANOVA, was also compared by using the mixed model ANOVA for repeated measures.

### 2.7. Morphology

When the end point was manifested, mice received deep chloral hydrate anesthesia. They were perfused transcardially by using a fixing solution composed of 150 mL 4% paraformaldehyde in PBS 0.1 N, pH 7.3, which was delivered by a peristaltic pump after washing with 50 mL of saline. After perfusion, the whole mice column was dissected and moved overnight to a solution of 4% paraformaldehyde for 24 h at 4°C. The spinal cord was carefully dissected from the column. From each mouse, a few mm piece from the lower lumbosacral tract of the cord was isolated to be further processed for semithin sections.

### 2.8. Light Microscopy

The spinal cord, except the small sample for semithin sections and further ultrastructural studies, was washed out in PBS and transferred into 70% alcohol solution at 4°C. All samples were dehydrated by increasing alcohol solutions, immersed in xylene for several hours, and finally embedded in paraffin. Seven *μ*m thick microtome transverse sections were collected serially. The thickness of the slices was planned to count two cell sizes roughly distinguishable around the central canal and within lamina X. This stereological-like procedure follows what previously was published to adapt cell count to heterogeneity of cell size of a given region [28], which was adapted here to the lamina X of the spinal cord. In detail, to identify lamina X, we used a mouse spinal cord atlas [25] referring to lumbar plates where the count was eventually carried out. We found that within lumbar lamina X including the central canal two predominant cell sizes ranging around 7 *μ*m and 20 *μ*m can be described. This preliminary evaluation of the cell diameters was determined at 40x magnification, using software for image analysis. When counting larger cells we measured the number of lamina X cells in one out of four sections, to provide a space interval of about 30 *μ*m. On the other hand, ependymal cells, with a smaller diameter (6-7 *μ*m), were counted in one out of two sections, to provide a space interval of about 14 *μ*m. A total of 400 sections/mouse per group were counted. Different cell sizes were cumulated in the final count. These counts globally refer to a tract of the lumbar cord 1.5 cm long.

Slices were stained with haematoxylin and Eosin (H&E) or against various antigens by using immunohistochemistry counterstained with haematoxylin (or DAPI for immunofluorescence).

Counts represent the number of cells counted in 1.5 cm of the lumbar cord and are expressed as the mean ± SEM for each group. Cell counts were performed by at least two different observers, unaware of treatments. Comparisons between groups were made by using a one-way analysis of variance ANOVA combined with Scheffè post hoc tests. Null hypothesis was rejected for *P* ≤ 0.05.

### 2.9. Immunohistochemistry

We used primary antibodies against nestin for undifferentiated, stem-like cells, the glial fibrillary acidic protein (GFAP), the early neuronal marker *β*III-tubulin, and the late neuronal marker NeuN. We also used primary antibodies against calbindin-D28K since this antigen is strongly expressed in the CNS during lithium-induced differentiation of neural progenitor cells [18–20, 32].

Slices were dewaxed by xylene, rehydrated by decreasing alcohol solutions, and permeabilized by Triton X 0.1% in PBS.

For immunoperoxidase, slices were preincubated with 3% hydrogen peroxide for 30 minutes to inhibit the activity of the endogenous peroxidases followed by a blocking solution (10% normal goat serum in PBS) for 1 hour at room temperature. Primary antibody solutions were prepared in PBS containing 2% normal goat serum and they were incubated overnight at 4°C. Primary antibodies were used at the following concentrations: mouse anti-*β*III-tubulin (1 : 50, Millipore, Billerica, MA, USA), mouse anti-GFAP (1 : 400, Sigma), mouse anti-NeuN (1 : 50, Millipore), and mouse anticalbindin-D28K (1 : 100, Sigma). The reaction with primary antibody was revealed by anti-mouse biotinylated secondary antibody (Vector Laboratories, Burlingame, CA, USA) which was used at a dilution of 1 : 200, for 1 hour, at room temperature. This was followed by incubation with avidin-biotin kit (Vector Laboratories), for 1 hour, at room temperature. The binding was revealed by using the peroxidase substrate diaminobenzidine (Vector Laboratories) for 1-2 minutes. All sections, except those labelled with anti-NeuN and anti-nestin antibodies, were counterstained with haematoxylin. Finally, sections were dehydrated by increasing alcohol solutions, clarified in xylene, and coverslipped with the mounting agent DPX (Sigma).

For the immunofluorescence slices were exposed to a mouse anti-nestin (1 : 200, Abcam, Cambridge, UK) primary antibody which was further revealed by using the anti-mouse fluorescent secondary antibody Alexa Fluor 488 (1 : 200, Life Technologies, Carlsbad, CA, USA). After washing in PBS, slices were coverslipped with Fluoroshield (Sigma). The nuclear dye DAPI (1 : 1000) (Sigma) was added to the solution to visualize cell nuclei. DAPI- and nestin-stained pictures were merged.

All stained slices were observed using a Nikon Eclipse 80i light microscope equipped with digital camera and software for image analysis.

### 2.10. Semithin Sections

In order to provide detailed representative images of ependymal cells we carried out semithin sections which were also used as a reference for further electron microscopy studies. Briefly, samples from lower lumbosacral segments previously fixed overnight 4% paraformaldehyde were dissected from spinal cord and switched to a 2% paraformaldehyde/0.1% glutaraldehyde in 0.1 N PBS, pH 7.4, for 90 min at 4°C. After washing samples were postfixed in 1% OsO_4_ in PBS, dehydrated in ethanol, and embedded in Epon-araldite. For identification of lamina X and ependymal area, 1-2 *μ*m thick serial sections, obtained with a porter blum MT-1 or an ultramicrotome Reichert-Jung, were stained with 1% toluidine blue and 1% methylene blue in 1% sodium tetraborate and observed under light microscopy.

## 3. Results and Discussion

### 3.1. Survival and Behaviour

**Figure 1:**
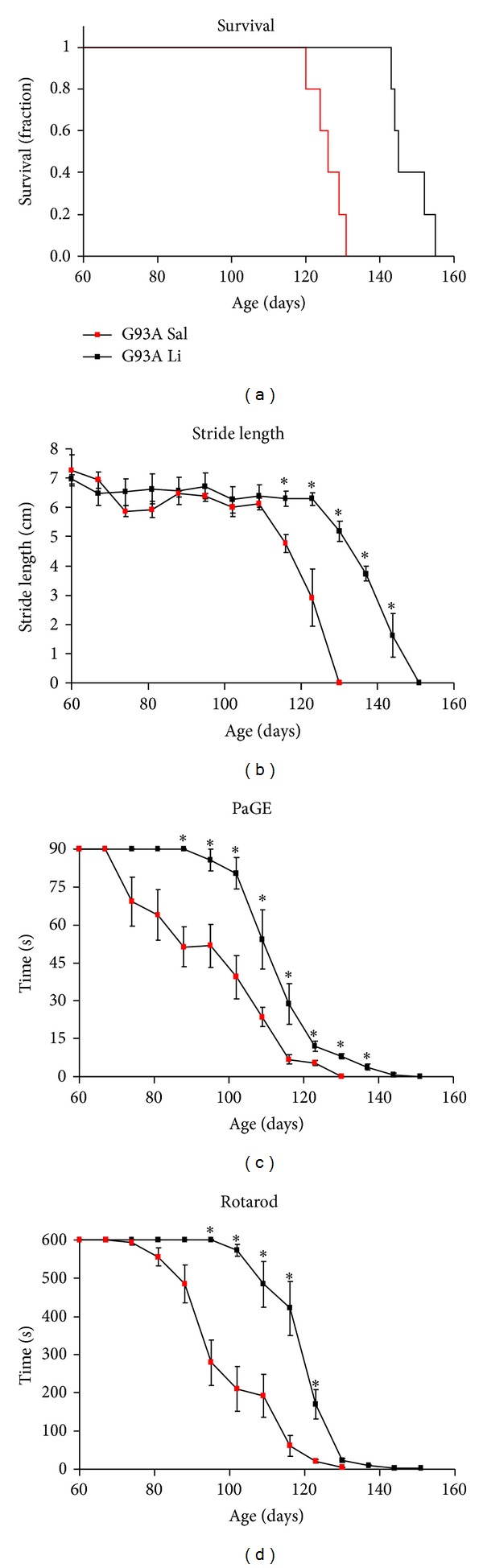
Effects of lithium on survival and motor performance in G93A mice. The graphs report data from G93A mice with either saline or lithium. Lithium prolongs mice survival as shown by Kaplan-Meier survival curve. Lithium counteracts motor deterioration measured by stride length, paw grip endurance (PaGE), and rotarod. Values are given as the mean ± SEM. *P ≤ 0.05 compared with mice administered saline.

As firmly established the G93A mutation of SOD1 led to a rapidly progressing palsy which was lethal in a few weeks after symptoms onset (Figure 1). As previously reported [19, 20, 28, 33, 34], chronic lithium administration prolonged significantly the survival as shown in the Kaplan-Meier curve (Figure 1), while it improved motor performance of G93A mice in all the motor tests (Figure 1). The behavioral deterioration and death were not abolished by lithium. Lithium was delaying the motor deterioration and prolonging the lifespan of the mice. These effects are evident more at the beginning compared with the end stage of disease, when these effects were no longer detectable. When we implemented statistical analysis by using mixed model ANOVA for repeated measures the protective effects of lithium on the decay of motor activity remain significant for a large part of the disease duration, but they were no longer significant in the last week before the end stage of disease (stride length and rotarod).

Protective effects of lithium in motor neuron disorders are documented in a variety of experimental conditions [19, 20, 28, 33–37]. Similarly, it was shown that lithium protects against neurotoxicity to peripheral axons [38, 39]. Nonetheless, a lack of effects of lithium in the G93A mouse model was also reported [40]. The discrepancy brought by this latter study may due to the fact that, for unknown reasons, authors did not administer lithium at levels required to produce its pharmacological effects such as autophagy induction [41]. As reported by Chiu et al. [42], it is likely that this depends on lithium concentrations achieved by Pizzasegola et al. [40] which are way below (at least 10 fold) those required to produce any pharmacological effect.

In the present study, in experimental conditions where lithium did provide neuroprotection, we analyzed the plastic effects induced by chronic lithium administration within lamina X.

### 3.2. Plasticity within Lamina X of the Spinal Cord during Motor Neuron Disease with or without Lithium Administration

**Figure 2:**
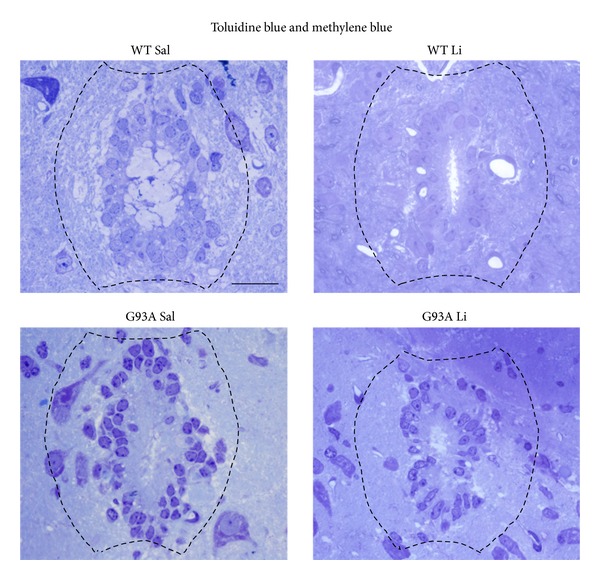
Pilot light microscopy procedures showing semithin sections. For identification of lamina X and ependymal area, 1-2 *μ*m thick serial sections, obtained with a porter blum MT-1 or an ultramicrotome, were stained with 1% toluidine blue and 1% methylene blue in 1% sodium tetraborate and observed under light microscopy. In each experimental group (wild type administered saline, WT Sal; wild type administered lithium, WT Li; transgenic SOD1 G93A mice administered saline, G93A Sal; transgenic SOD1 G93A mice administered lithium, G93A Li) the central canal is limited by densely packed cells with polymorphic nuclei, which increase their number and appear as multiple call layers in G93A mice compared with WT. Again, in G93A mice, the staining is consistently more intense compared with WT. Scale bar = 21 *μ*m.

**Figure 3:**
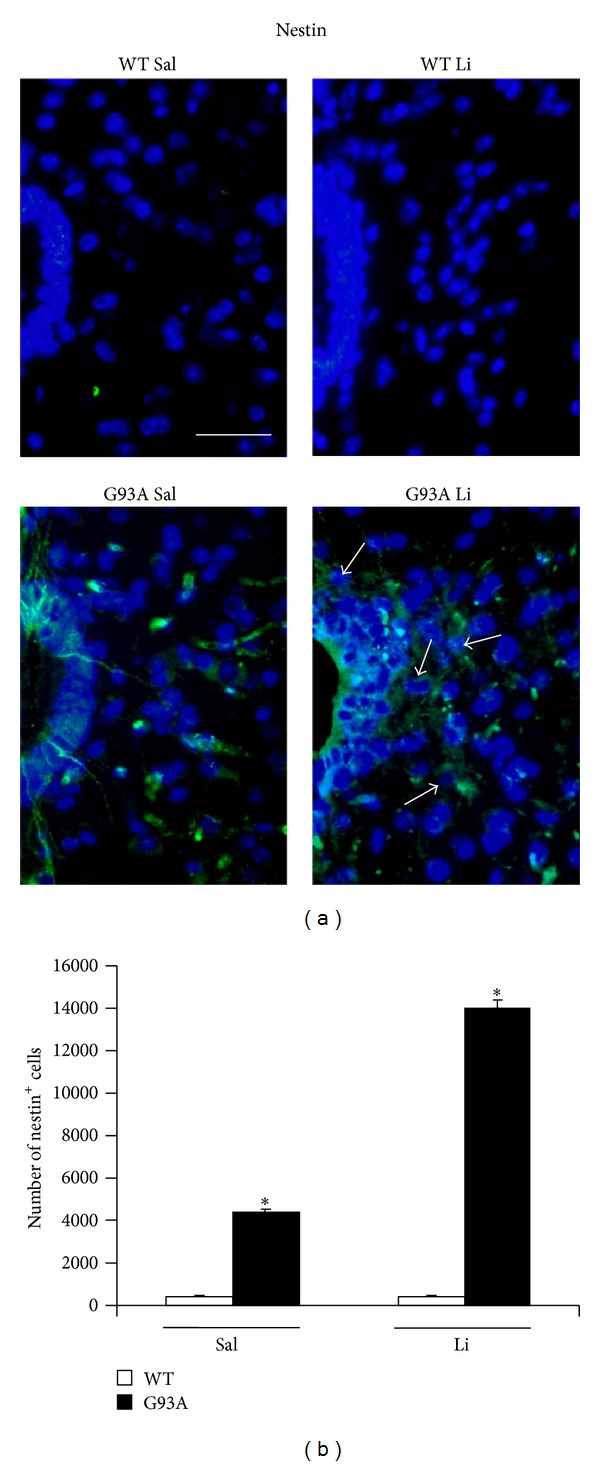
Nestin immunostaining within lamina X. Representative pictures are reported in (a) showing that lamina X shows neglectable nestin-immunofluorescence in WT mice (both WT Sal or WT Li). In contrast, nestin immunostaining is well evident within the lamina X from ALS mice (both G93A Sal and G93A Li). In particular, in G93A mice treated with saline nestin-positive fibers between the ependymal cells appear to project from the ependymal canal into lamina X, where only a few cells appear labelled. In contrast, in G93A mice treated with lithium nestin immunofluorescence is dramatic and involves both the ependymal cells and stem-like cells within the entire lamina X (arrows). (b) The graph reports the count of nestin-positive cells in both lamina X and ependymal canal. *P ≤ 0.05 compared with other groups. Scale bar = 26 *μ*m.

**Figure 4:**
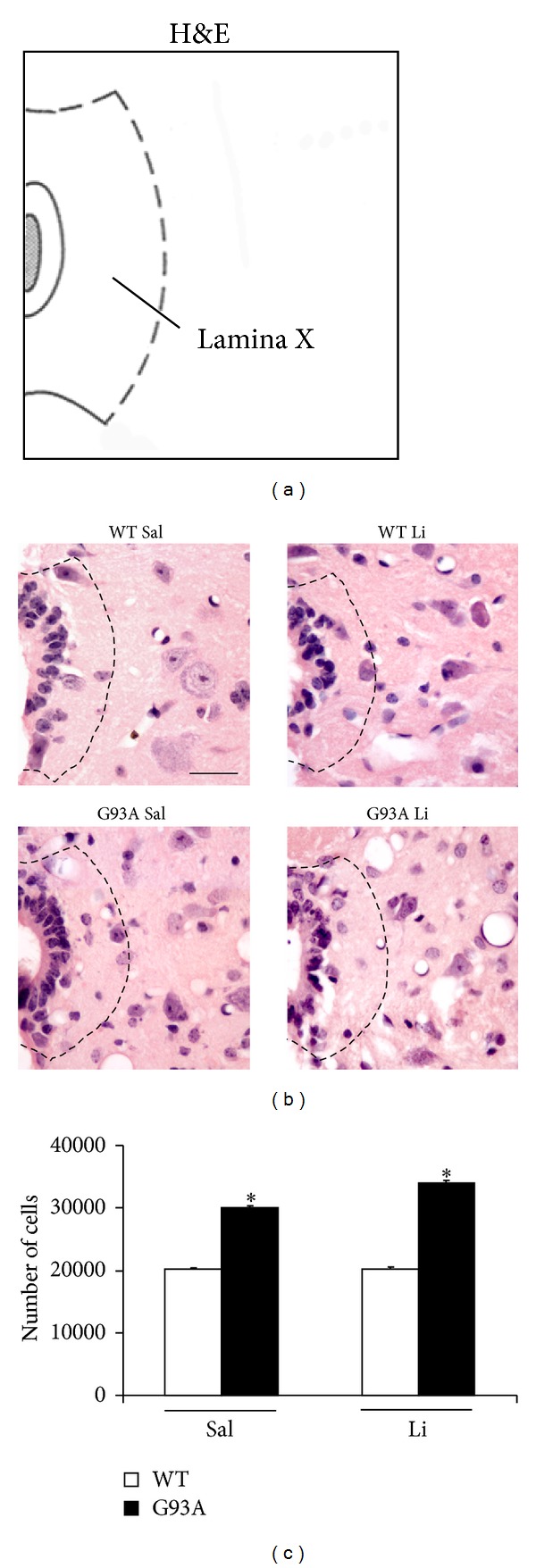
H&E staining of lamina X. (a) Atlas plate [25] of the lumbar cord (L_4_) used as the reference for representative pictures. (b) Representative high magnification of H&E stained pictures showing L_4_ lamina X, treated with either saline or lithium. The cell number counted within lamina X is equivalent for WT mice treated with either saline (WT Sal) or lithium (WT Li). On the other hand, the cell number is increased in the lamina X of G93A mice treated with saline (G93A Sal) or lithium (G93A Li). When counting the cell number it is evident that, as reported in the histogram (c), the cell number in lamina X is mostly increased in G93A mice receiving lithium. The increase in the cell number occurring within lamina X of G93A mice is better visualized at the level of the ependymal layer. Dashed lines draw the border of the lamina X. *P ≤ 0.05 compared with other groups. Scale bar = 30 *μ*m.

**Figure 5:**
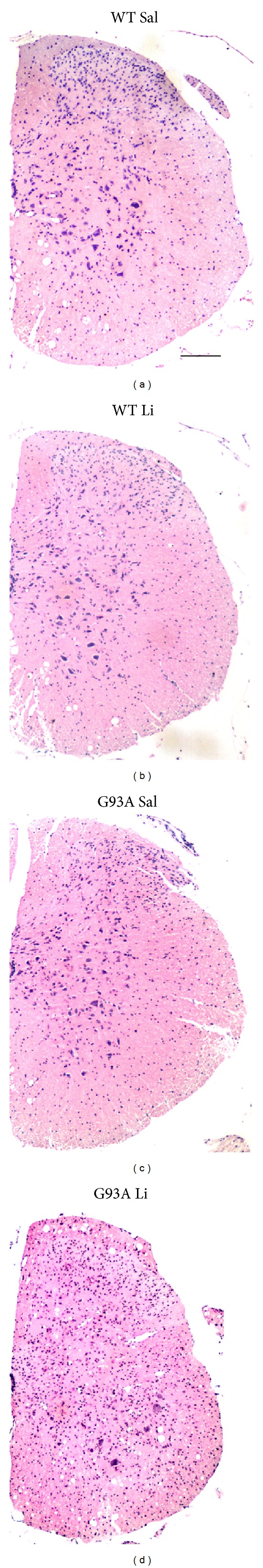
Representative pictures from hemi-lumbar cord. Representative pictures of H&E stained hemi-lumbar cord. In the upper lane are reported pictures from wild type mice treated with either saline (WT Sal) or lithium (WT Li). In the lower lane are reported G93A mice treated with either saline (G93A Sal) or lithium (G93A Li). Pictures are representative of end stage of disease. Noteworthy is the generalized increase in spinal cord cell density which is produced by lithium administration in G93A mice. Such an effect appears to extend in the whole grey matter. Scale bar = 232 *μ*m.

**Figure 6:**
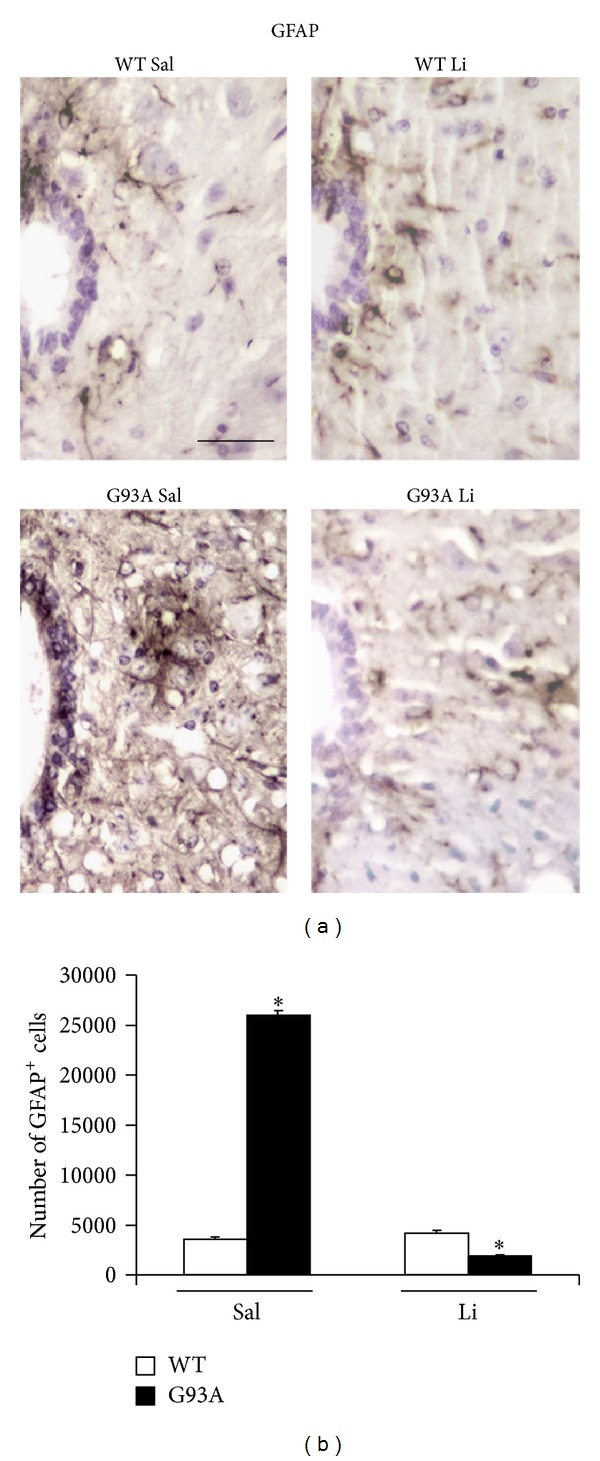
GFAP immunostaining within lamina X. Representative high magnification of GFAP immunostained slices from lamina X of lumbar spinal cord (a). The cell number counted within lamina X is equivalent for WT mice treated with either saline (WT Sal) or lithium (WT Li). On the other hand, the cell number is dramatically increased in the lamina X of G93A mice treated with saline (G93A Sal), while it is suppressed in G93A mice treated with lithium (G93A Li) as reported in the histogram (b). In general, GFAP immunostaining shows positive cells on external side of the ependymal layer, while in G93A Sal intense immunostaining extends in the surrounding lamina X. *P ≤ 0.05 compared with other groups. Scale bar = 30 *μ*m.

**Figure 7:**
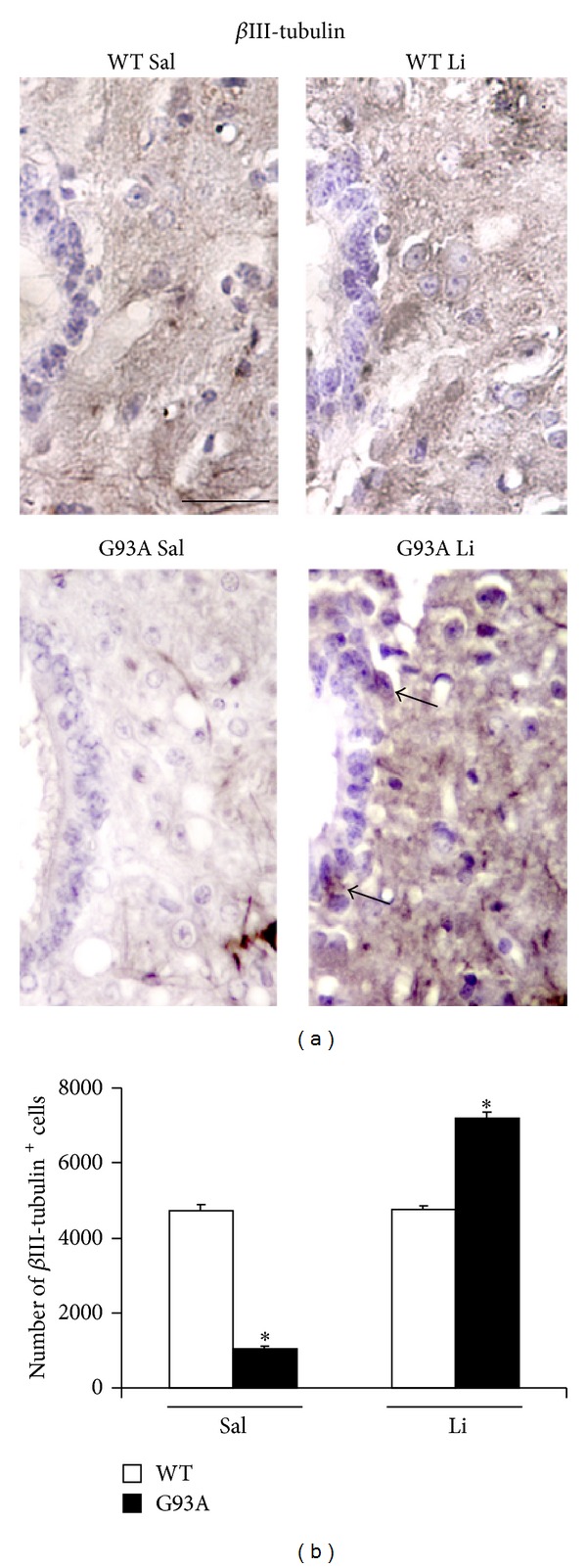
*β*III-tubulin immunostaining within lamina X. Representative high magnification of *β*III-tubulin immunostained slices from lamina X of lumbar spinal cord (a). The cell number counted within lamina X is equivalent for WT mice treated with either saline (WT Sal) or lithium (WT Li). On the other hand, the cell number is dramatically increased in the lamina X of G93A mice treated with lithium (G93A Li), while it is suppressed in G93A mice treated with saline (G93A Sal) as reported in the histogram (b). In general, *β*III-tubulin immunostaining shows an opposite pattern compared with GFAP (see Figure 6). Following lithium administration an intense immunostaining is visible throughout the whole lamina X of G93A mice. *P ≤ 0.05 compared with other groups. Scale bar = 30 *μ*m.

**Figure 8:**
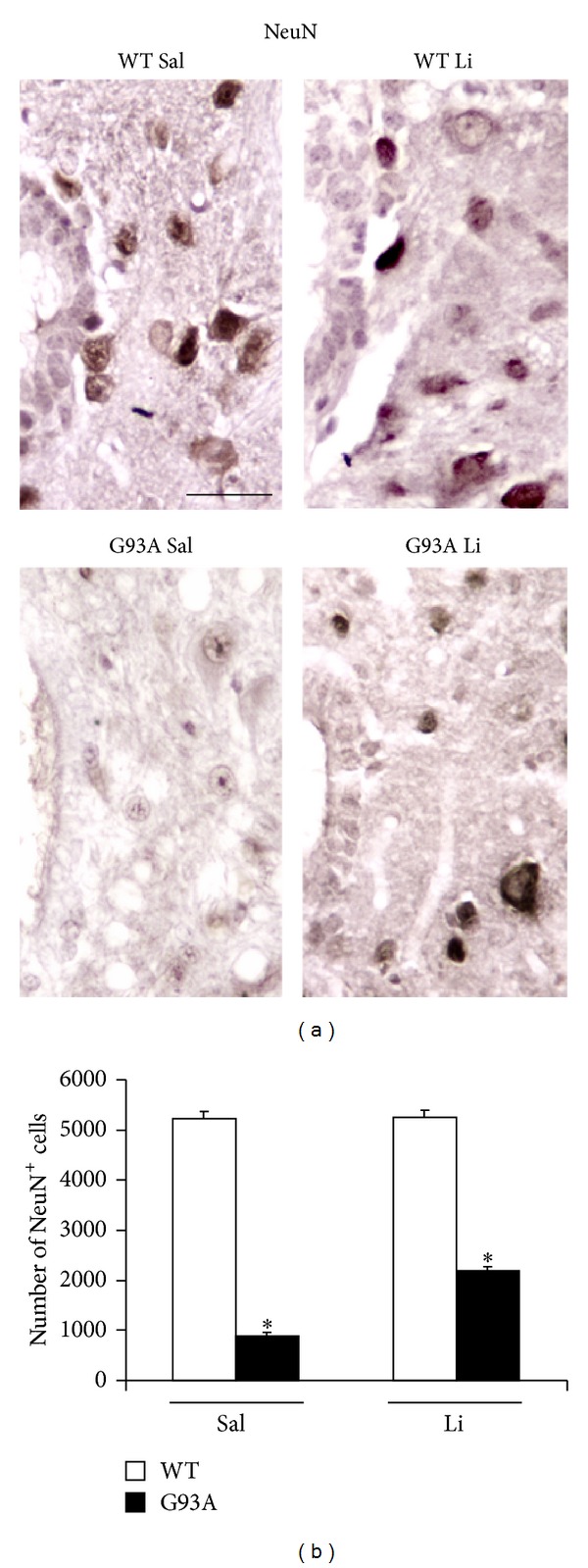
NeuN immunostaining within lamina X. Representative high magnification of NeuN immunostained slices from lamina X of lumbar spinal cord (a). Again, immunopositive cells within lamina X for WT mice treated with either saline or lithium (WT Sal or WT Li, resp.) is equivalent. On the other hand, NeuN positive cells are suppressed in the lamina X of saline treated G93A mice (G93A Sal), while lithium administration (G93A Li) partially recovers NeuN immunostaining as reported in the histogram (b). *P ≤ 0.05 compared with other groups. Scale bar = 30 *μ*m.

Figure 2 shows increased basophilic cell density around the central canal, which occurs selectively in G93A mice. This is in line with increased mitosis occurring in ALS spinal cord reported by Chi et al. [8]. Spinal cord plasticity based on stem cells proliferation within adult spinal cord is a hot topic under intense investigation, in relationship with both spinal cord injury and degenerative motor neuron disorders. A very recent state-of-the-art review [7] clearly indicated that, within spinal cord, adult stem cells originate from the ependymal layer within lamina X. This is based on genetic studies mapping the fate of these cells along with flow cytometric characterization [7, 22–24]. In baseline conditions stem cells from the spinal cord are rarely mitotic, while they increase dramatically their mitosis following injuries. These data are confirmed here by staining the stem cell antigen nestin. This was neglectable in WT, while it increased markedly in G93A mice. Noticeably, when G93A mice were administered with lithium nestin immunopostive cells further increased, as shown in Figure 3. This increased mitogenic activity in lamina X was confirmed by plain cell count following H&E staining as reported in Figure 4. Most of the studies analyzing spinal cord stem cells in disease states were focused on traumatic or other acute spinal cord injuries (for a review [7]), while only a few studies were carried out in the course of ALS. In line with the literature describing the stem cells fate following spinal cord injuries we already described a general increase in cell density in the lamina VII of spinal cord of ALS mice along with increase in BrdU staining [19, 20]. In the present study we counted an increased number of stem-like cells within lamina X of G93A mice (Figure 3) and a general increase in cell number in the same region of ALS mice (Figure 4). This occurs more abundantly in G93A mice treated with lithium as evident in representative Figure 4(b) and as reported in the counts of Figure 4(c). Moreover, when examining at low magnification the hemi-spinal cord from G93A mice a generalized increase in cell density is evident which also extends to the entire dorsal horn (Figure 5). The increase in stem-like cells and total cell number we measured in the lamina X is in line with the increase in NPC previously reported in ALS mice [8]. Such an increase (both for stem-like cells, Figures 3(a) and 3(b), and total cells, Figures 4(b) and 4(c), resp.) is more evident when G93A mice were administered with lithium. Lithium alone, administered to WT mice, did not increase neither nestin-positive cells nor total cell number. In the paper by Chi and collaborators [8] it was described that most of the NPC occurring in the ALS spinal cord differentiate towards glia, which is the case also following spinal cord injury [7]. In fact, in the present study, we found a dramatic increase in GFAP immunopositive cells in the lamina X of G93A mice administered with saline (Figures 6(a) and 6(b)). In sharp contrast, the amount of GFAP positive cells in G93A mice administered with lithium were suppressed even compared with controls (Figures 6(a) and 6(b)). These latter findings brake the concept of the gliogenetic fate of stem cells in the spinal cord. So far, increased expression of GFAP occurs constantly when spinal cord stem-like cells are proliferating following either spinal cord injuries [7, 23] or ALS [8]. In sharp contrast, in the present study, we documented that, in G93A mice, chronic lithium administration produces a dramatic increase in nestin immunostaining but suppresses GFAP immunostaining (Figures 3 and 6, resp.). Such a switch in cell differentiation is further substantiated by the dramatic increase in early and late neuronal markers *β*III-tubulin (Figures 7(a) and 7(b)) and NeuN (Figures 8(a) and 8(b)) occurring when lithium was administered to G93A mice. This is opposite to what occurred in saline administered ALS mice owing to increased GFAP positive cells with no increase in neuronal markers within lamina X (*β*III-tubulin, Figures 7(a) and 7(b), and NeuN, Figures 8(a) and 8(b)). Thus, the gliogenic effects which routinely characterize increased nestin-positive cells in the diseased spinal cord were reversed here by chronic lithium administration, which increases neuronal markers while suppressing GFAP positive cells. In WT mice lithium did not produce any noticeable change in nestin, GFAP, *β*III-tubulin, or NeuN immunostaining. This is in line with the concept that proliferation of ependymal cells in the spinal cord is scarce in baseline conditions. Only spinal cord injury [23, 43, 44] or degenerative disorders [8, 19] produce a stimulation of neurogenesis in the ependymal zone of lamina X. Lithium magnifies such an effect by further increasing cell number and, most importantly, addressing their differentiation towards a neuron-like phenotype while inhibiting gliogenesis. These effects were described following adult spinal cord injury, and now they extend to a chronic model of motor neuron disorder.

**Figure 9:**
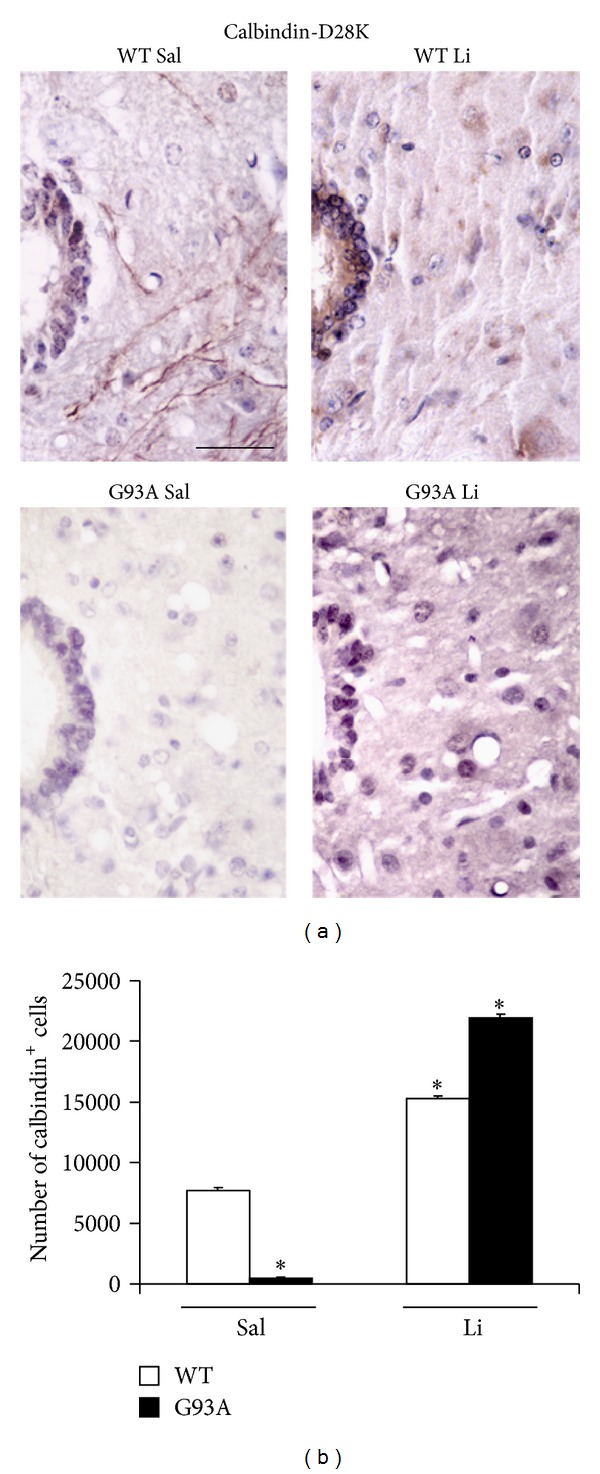
Calbindin-D28K immunostaining within lamina X. Representative high magnification of calbindin-D28K immunostaining within lamina X of lumbar spinal cord (a). In general, intense calbindin-D28K immunopositivity appears to be localized in the ependymal layer. The cell number counted within lamina X is markedly increased by lithium in both WT and G93A mice (WT Li and G93A Li, resp.). On the other hand, the cell number is reduced in G93A mice administered saline (G93A Sal). This latter effect is particularly evident in the area of lamina X external to the ependymal canal. In this area only pale cell shapes avoided from the count are visible, which explains the very low cell number reported in the histogram (b). In G93A mice the effects of lithium extend the strong immunostaining to the entire lamina X. *P ≤ 0.05 compared with other groups. Scale bar = 36 *μ*m.

In fact, by using the G93A mouse model of ALS, here we provide specific evidence that lithium increases total cell number in the lamina X of the spinal cord (Figure 4), thus confirming previous experimental observations [20]. In detail, in the present paper, we found that the disease itself increases the cell number within lamina X of the spinal cord (Figure 4). This effect is magnified by lithium administration, only in G93A mice (Figure 4). When lithium was administered to WT no increase in the total lamina X cell number was observed (Figure 4). Interestingly, this confirms what previously reported [8] showing a compensatory activation of neurogenesis in the spinal cord during ALS. Such a confirmation was directly measured in the present work by counting the number of nestin-positive cells (Figure 3). However this neurogenetic effect is routinely expected to lead to glia [8]. This was also confirmed in the present work, where in G93A mice administered with saline an increase in GFAP positive cell number was detected within lamina X (Figure 6). However, this was reversed by the administration of lithium, which suppressed the number of GFAP positive cells (Figure 6) while markedly increasing the number of positive cells for the early or late neuronal marker *β*III-tubulin (Figure 7) and NeuN (Figure 8) in G93A mice. In detail, while increased cell number occurs in the lamina X of G93A mice independently by the administration of lithium, it is just the presence of lithium which addresses these cells towards a neuron-like phenotype instead of glia. This effect reminds what it is well established for the effects of lithium in the hippocampus and SVZ where it induces neurogenesis while inhibiting glial differentiation and promoting neuronal differentiation [18, 45–56]. In fact, it is well known that lithium in the hippocampus specifically induces the occurrence of calbindin-D28K positive neuron-like cells [18]. Remarkably, we found here that even in the spinal cord lamina X lithium promotes the increase of calbindin-D28K positive neurons, which occurs in both G93A mice and WT (Figure 9).

Lamina X is known to be a niche for stem cells in the adult spinal cord [57]. In our hands such a niche appears to be “lazy” meaning that it is dormient in baseline conditions and it reacts with cell proliferation without neuronal differentiation in disease conditions. The concomitant administration of lithium in disease conditions enhances cell proliferation and addresses them towards a neuron-like phenotype. The occurrence of proliferating nestin-positive stem-like cells described here confirms the occurrence of actual cell mitosis following lithium in ALS spinal cord which we already described by merged BrdU and calbindin-D28K immunostaining [19].

It is interesting that lithium administration needs to be carried out in G93A mice in order to produce an increase in neurogenesis and neuronal differentiation in the spinal cord. In fact, lithium by itself does not produce any increase in the number of nestin-, *β*III-tubulin-, and NeuN-immunopositive cells. Moreover, even the number of H&E stained cells does not increase in WT mice administered with lithium. In contrast, lithium alone (in WT mice) is sufficient to increase the number of calbindin-D28K positive cells. This suggests that this specific effect may be the consequence of a mere phenotypic shift. This hypothesis is consistent with the increase of calbindin-D28K positive cells which was more marked compared with BrdU positive cells in the spinal cord of G93A mice [19].

The activation of the spinal cord ependymal niche is recently described by other authors [57] who found a pattern of radial nestin immunostaining occurring after a variety of disorders of the spinal cord which is reminiscent of what we observed in representative pictures (Figure 3(a)). In line with these studies we found that nestin immunopositive cells possess radial processes and, as happening after spinal cord injury, they are more abundant in the lateral aspects of the canal. Altogether, these data provide novel evidence on plastic phenomena detectable at the level of the spinal cord niche in the lamina X during an ALS model and substantiate the modulatory effects of lithium on these phenomena. It is exciting that these data replicate what was already found more profusely for the effects of lithium in the telencephalon at the level of SVZ. Again, these data extend to amyotrophic lateral sclerosis which was previously reported following spinal cord injury [52, 58] showing the prosurvival effects produced by lithium administration for the neuron-like stem cells either autonomous or implanted in the damaged cord. This might explain very recent data confirming the neuroprotective effects of lithium also against spinal cord injury [59].

## 4. Conclusions

The present experimental work, apart from providing a confirmation of the protective effects of lithium in the course of the G93A mouse model of ALS, it shed new lights on the plastic phenomena ongoing in this experimental model of motor neuron disease and their modulation induced by the mood stabilizer lithium. Interestingly, most of these plastic effects are disease dependent, while other are lithium dependent and most of them are enhanced by the administration of lithium in disease conditions. This is the case of the increase in nestin-positive cells around the canal, as well as the increase in NeuN and *β*III-tubulin positive cells. Thus, as shown for spinal cord trauma, lithium is likely to be a powerful modulator for stem cell differentiation also in ALS. In summary, in the present work we found that, in the presence of a chronic spinal cord disorder (ALS-alike G93A mice), the ependyma-containing lamina X appears to be enriched of newly proliferating cells which is reminiscent of what happens following a spinal cord injury. As described following a spinal cord injury, these cells appear to differentiate towards a glial phenotype. The most remarkable plasticity we described here consists in the combined effects of lithium administration in a spinal cord disorder. In fact, in these specific conditions lamina X cells appear as neuron-like cells expressing early and later neuronal markers along with calbindin-D28K, thus being reminiscent of the active stem cell niche in the SVZ under the effects of lithium.

These findings shed new light on what Sabelström et al. [7] just posed as an apparent limitation of spinal cord stem cells, that is, the tendency to produce solely a glial phenotype. In fact these authors, based on data obtained from diseased spinal cord (mostly traumatic), concluded literally that “the spinal cord microenvironment appears to have a strong gliogenic influence and/or fails to support neuronal survival.” This is in line with what we found in the natural course of ALS mice without lithium administration. Adding lithium is likely to address lamina X cells towards neuronal survival while suppressing the gliogenic influence. Further studies are needed to dissect the molecular mechanisms underlying the phenomena we formally described in the present paper. Moreover, beyond antigen expression, it is important to verify whether the effects of lithium on the ALS spinal cord produce electrophysiological effects which substantiate the neuronal phenotype. Finally it would be worthy to investigate the fate of the neuron-like cells. Do they synaptically integrate in the spinal cord under disease? Do they represent a paracrine source of active cytokines? Or do they merely represent an inert biological entity? We like to finish the conclusions with these big open questions.
